# Costs and implementation outcomes associated with strategies to increase use of a hospital assisted mobility program: evidence from a hybrid type 3 cluster randomized trial

**DOI:** 10.1186/s43058-026-01054-2

**Published:** 2026-08-08

**Authors:** Connor Drake, Cassie Meyer, Karen M. Stechuchak, Brystana G. Kaufman, Amy Webster, Caitlin B. Kappler, Ashley Choate, Leah L. Zullig, Jaime Hughes, Kelli D. Allen, Courtney H. Van Houtven, S. Nicole Hastings

**Affiliations:** 1 Center of Innovation to Accelerate Discovery and Practice Transformation (ADAPT), Durham VA Medical Center, 411 W Chapel Hill St, Suite 600, Durham, NC 27701 USA; 2 Department of Population Health Sciences, Duke University School of Medicine, Durham, NC USA; 3 Duke Margolis Institute for Health Policy, Duke University, Durham, NC USA; 4 Wake Forest University School of Medicine, Winston-Salem, NC USA; 5 Department of Medicine, University of North Carolina at Chapel Hill School of Medicine, Chapel Hill, NC USA; 6 Department of Medicine, Duke University School of Medicine, Durham, NC USA

**Keywords:** Implementation cost, Implementation strategies, Hospital mobility program

## Abstract

**Background:**

Assessing the cost of implementation strategy bundles and the relationship between costs and implementation outcomes is critical for informing investments in spreading an evidence-based practice. Thus, we compared costs of two strategies with different levels of intensity to support implementation of ‘assiSTed eaRly mobIlity for hospitalizeD older vEterans’ (STRIDE), an evidence-based mobility program that consists of a gait-assessment and supervised walks for hospitalized older adults in the Veterans Health Administration (VHA).

**Methods:**

We measured STRIDE implementation costs across 35 VA medical centers (VAMC). As part of a type 3 hybrid cluster randomized trial, VAMCs were randomly assigned to receive either (i) standardized technical support (REP) or (ii) standardized technical support and external practice facilitation (enREP). We used an activity-based costing methodology to account for costs for external and site-level implementation activities across a 10-month active implementation period from the perspective of health system leadership. Costs were obtained using survey data and national salary estimates. We describe the association between implementation costs and STRIDE penetration, or the extent to which the program reached patients, and program adoption, based on reaching a pre-specified level of program activity.

**Results:**

Median REP VAMC (*n* = 19 VAMCs) cost was $18,645 and varied significantly (Q1–Q3: $14,089–$28,579) and median enREP VAMC (*n* = 16 VAMCs) cost was $20,007 (Q1–Q3: $17,341 - $23,083). 10% of REP costs and 13% of enREP costs were associated with external support. Implementation cost was not strongly correlated with penetration of STRIDE (Spearman *r* = 0.08). Costs were greater at REP VAMCs that adopted STRIDE. For enREP VAMCs, costs were similar for VAMCs that did and did not adopt STRIDE.

**Conclusions:**

The enREP intensified facilitation support resulted in an increase in cost compared to REP but was not associated with improved implementation outcomes. Decision-makers planning to scale STRIDE-like hospital mobility programs should anticipate that local site-level costs are the largest driver of cost and should be the principal target for budget planning. Study findings may have implications for how to best design and deploy varying levels of intensity of implementation support to effectively scale hospital mobility programs in real-world care settings.

**Trial registration:**

ClinicalTrials.gov (identifier NCT04868656). No key protocol deviations or unplanned analysis related to cost occurred in this trial.

**Supplementary Information:**

The online version contains supplementary material available at https://doi.org/10.1186/s43058-026-01054-2.

Contributions to the literature • The negative consequences of immobilizing of older adults while hospitalized are known. Evidence based assisted mobility programs, like STRIDE (ASsisTed EaRly MobIlization for hospitalizeD older VEterans), can improve outcomes and control cost. STRIDE is a low-cost intervention; however, little is known about the cost or efficiency of implementation strategies to support its use.

• This study uses an activity-based costing methodology to assess the cost of two approaches to support implementation of STRIDE: a standardized, tested bundle of implementation strategies based on the Replicating Effective Programs (REP) and a more tailored and intensified approach that also included practice facilitation called Enhanced REP (enREP).

• Implementation trials rarely report detailed cost analyses of the strategies tested resulting in an evidence gap for decision-makers investing in scaling a novel, evidence-based program or practice. This study’s methodological approach may serve as a useful exemplar for performing and reporting cost analyses alongside implementation outcomes to assess the efficiency and value of strategies.

## Introduction

The translation of novel, evidence-based programs (EBPs) into real-world clinical settings is costly and complex, but critical to improving health outcomes. Understanding the most efficient implementation strategies to support adoption, reach, and sustainability of EBPs is needed to make informed decisions on how to adequately resource implementation efforts [1, 2]. However, a systematic review of the cost of implementing evidence-based guidelines found that there were very few economic evaluations and, of those published, their impact is limited due to methodological limitations [3]. This study builds evidence by conducting an implementation costing study of two strategies to support implementation of ‘assiSTed eaRly mobIlity for hospitalizeD older vEterans’ (STRIDE), an evidence-based mobility program for hospitalized older adults in the Veterans Health Administration (VHA).

The VHA is a learning laboratory for testing national implementation strategies for EBPs because it is the largest integrated health system in the United States, serving approximately 10 million Veteran households, and has an organizational commitment to continuously improving quality for Veterans and their families [4]. This includes a VHA system-wide focus on reducing the functional decline of older Veterans by promoting an ‘Age-Friendly Health System’ [5]. Hospitalized older adults often experience prolonged immobility and resultant hospitalization-association disability and functional decline after their inpatient stay [6–8]. It is common for hospitalized older adults to have a new disability that was not present before they were hospitalized, resulting in poor outcomes that accelerate the functional decline of older adults [9, 10]. To mitigate the negative consequences of immobility, the VHA is committed to implementing an evidence-based inpatient supervised walking program, STRIDE (assiSTed eaRly mobility for hospitalizeD older vEterans), system-wide [11].

STRIDE has been endorsed as a VHA best practice and consists of a one-time gait and balance assessment and supervised daily walks throughout the duration of the hospitalization. STRIDE, and inpatient mobility programs like it, can improve health outcomes and reduce health care costs by reducing patients’ length of stay, improving the likelihood of being discharged to home, and mitigating functional decline [12, 13]. Further, STRIDE intervention costs are modest (<$30 per patient) [14]. However, introducing STRIDE to a new hospital requires complex and novel practice patterns and interdependencies between existing clinical teams and typically occurs over 6–10 months [9, 15]. To ensure timely and efficient translation of STRIDE, strategies were developed to support its successful implementation using a rigorous, shareholder-engaged process [16, 17].

This hybrid type 3 trial, meant to evaluate the success of an implementation strategy as its primary goal, was designed to assess the comparative effectiveness of the REP and enREP strategies to enhance STRIDE penetration, among other implementation outcomes of interest, and included survey data collection to assess the direct costs associated with the implementation effort. We conducted a hybrid type 3 implementation-effectiveness trial to compare the impact of two bundles of implementation strategies based on the Replicating Effective Programs framework: REP and enREP [18]. The bundles varied in intensity, with the enREP strategy including a higher intensity approach that featured practice facilitation, an evidence-based approach that has demonstrated a positive impact on implementation outcomes [19], including research at the VHA [20]; however, it is a more labor-intensive and, therefore, a costly strategy to deploy at scale. Thus, understanding REP and enREP cost and differential impact on implementation outcomes will be critical for understanding the efficiency of an investment in an intensified approach.

Few studies conduct comparative analyses of implementation strategies [2, 3, 21]. Further, inconsistent methods for reporting cost estimates and analysis of ‘return-on-investment’ presents a barrier to evidence generation [21]. This is a persistent challenge for health care leaders who are making decisions regarding deploying implementation strategies without information on the potential cost or calibrated expectations of the ‘return-on-investment’ in terms of promoting the use of a novel EBP [1].

This study has the following objectives: (i) to estimate the cost of the REP and enREP implementation strategy bundles at the diverse participating VAMCs; (ii) to estimate variation of implementation costs across the active implementation period; and (iii) to describe the relationship between the cost of implementation strategies with implementation outcomes of interest: penetration and adoption. By answering these questions, we aim to address evidence gaps that impede our understanding of how to adequately resource implementation of the STRIDE evidence-based hospital mobility program.

## Methods

### Primary study design overview

**Fig. 1:**
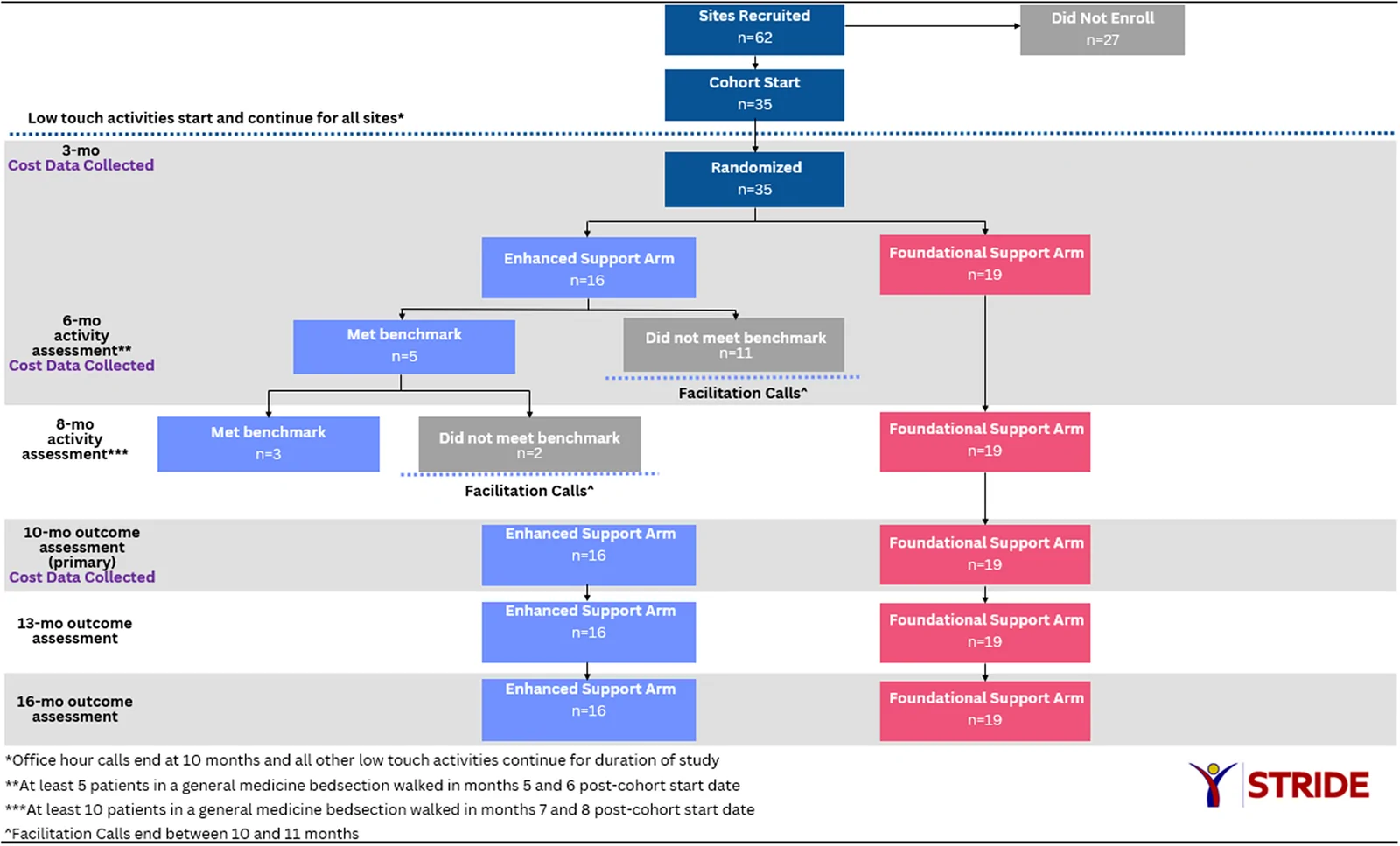
Function QUERI trial design and data collection overview. *≥5 patients in a general medicine bedsection received STRIDE in months 5 & 6; **≥10 patients in a general medicine bedsection received STRIDE in months 7 & 8

Data were derived from Function QUERI, the Optimizing Function and Independence QUERI trial (ClinicalTrials.gov, NCT04868656. Registered on June 01, 2021). Function QUERI included a hybrid type 3 cluster randomized controlled trial that compared the effectiveness of two implementation strategy bundles based on the Replicating Effective Programs (REP) framework to improve STRIDE implementation: 1) REP and 2) enREP. Veteran Affairs Medical Centers (VAMCs) were enrolled in five successive cohorts every 3 to 4 months from June 1, 2021, to October 3, 2022. Stratified block randomization was used to randomize hospitals using equal allocation strategy (1:1) to either REP or enREP. Additional detail on Function QUERI trial design and the bundle of implementation strategies is published elsewhere [22] and presented in Fig. 1.

Evaluation activities were approved as human subjects research by the Durham VA Institutional Review Board. The study is reported according to the 2022 Consolidated Health Economic Evaluation Reporting Standards 2022 (CHEERS) for cost analyses (See ‘Additional file 1’ for the completed CHEERS checklist).

### Study setting and patient population

Hospital eligibility criteria included: (i) leadership support and willingness to implement STRIDE; (ii) voluntarily receive implementation support according to their randomization assignment; (iii) to deliver STRIDE with their own clinical resources. Participating VAMC were advised on the core components of STRIDE designed for patients 60 years old and over, the demographic at highest risk for functional decline due to immobility in the hospital [23]. Eligible hospitals were identified and invited to participate through several mechanisms including recruitment calls, word of mouth, operational partner referrals, and utilizing Microsoft Identity Manager to identify potential points of contact (POCs) at hospitals. Additional details on the site identification and recruitment process are published elsewhere [24].

STRIDE is an evidence-based mobility program for hospitalized older adults aimed to enhance functional independence. STRIDE consists of a targeted gait and balance assessment followed by daily supervised walks throughout the patient’s hospital stay. In previous trials, STRIDE has shown to reduce hospital length of stay and a reduced discharge to skilled nursing facilities [12].

### Implementation strategies

The REP framework has been described as both as an implementation framework and strategy for supporting implementation of an evidence-based practice and selected because of its prior validation [18, 24, 25]. We refer to REP as an implementation strategy bundle that consists of specific ‘low-touch’ activities designed to support implementation that are offered to all participating VAMC sites.

REP ‘low touch’ activities include the following five elements:

1. STRIDE Toolkit: Standardized program materials (e.g., webinars).
2. Digital Resources: Electronic health record documentation templates, training materials, marketing materials, and other standardized materials.
3. Data Dashboards: Access to a STRIDE report that lists potentially eligible patients and real-time program activity data using electronic health record data hosted by existing VA infrastructure to assist hospitals with tracking implementation.
4. Diffusion Network: Quarterly teleconferences with other STRIDE VAMC sites to provide continuous consultation and technical guidance on topics like documentation, staffing models, program adaptations, and to showcase best practices at different VAMCs.
5. Real-Time Chat Technical Assistance: Technical support and inter-site communication via Microsoft TEAMS infrastructure. This provides a forum for staff and clinicians from STRIDE to share best practices with minimal engagement from study team members.

VAMCs initially randomized to enREP that did not meet STRIDE program activity benchmarks at 6 (initial) or 8 (sustainment) months, respectively, transitioned to more intensive, tailored support featuring external facilitation via enREP. The overall active implementation period for both arms was 10 months. EnREP intensified from the implementation support provided in REP by incorporating practice facilitation, or a range of structured activities offered by a trained member of the study team who served as an external facilitator and offered support and project management during the active implementation effort consistent with evidence-based practice facilitation best practices. Facilitation activities included goal setting, identifying and assisting in resolving implementation barriers in real-time, providing monthly audit and feedback metrics, and providing input on technical aspects of the site-specific aspects of the implementation plan (e.g., the design of patient identification and referral workflows). External facilitators conducted a needs assessment to identify barriers and then led structured engagements or ‘working meetings’ with the VAMC-based teams which typically consisted of 2–3 clinicians and staff members with duties relevant to implementation activities (e.g., hiring, training, electronic health record integration, and establishing workflows and use of existing data dashboards). The external facilitation team consisted of both the trained facilitator and another study team member to take notes and monitor strategy fidelity for evaluation purposes. Since the other study team member was serving primarily in a research capacity, their effort was not included in activity-based costing estimates. Additionally, VAMCs receiving enREP had access to a VAMC-specific Microsoft TEAMS channel to connect them with other sites as part of a cohort of VAMC sites implementing STRIDE and the external facilitator. Each VAMC participated in a minimum of 4 synchronous audio/video facilitation meetings and site contact time did not exceed 6 hours over the ~4-month intensified support period. Additional detail on the implementation strategies and associated activities are reported in Table 1.

**Table 1:** REP and enREP implementation strategy activities

ERIC Implementation Strategy	Description	REP	enREP
Develop Educational Materials	STRIDE Toolkit: Provides standardized implementation materials, including recorded webinars and step‑by‑step guidance to support hospital teams in launching and maintaining STRIDE.	✔	✔
Distribute Educational Materials	Digital Resources: Offers access to a continually updated repository of EHR templates, training materials, and other standardized implementation documents to support consistent program delivery.	✔	✔
Develop and Organize Quality Monitoring Systems	Data Dashboard: Gives hospitals access to workload reports and lists of potentially eligible patients, enabling real‑time tracking of STRIDE activity and patient reach.		
Create a Learning Collaborative	Diffusion Network: Provides blended national and cohort‑level support through an initial Welcome Call, monthly cohort‑based Office Hours for progress updates and shared problem‑solving, and quarterly national calls to exchange best practices, receive technical guidance, and strengthen implementation and sustainment.	✔	✔
Provide Ongoing Consultation	Real-Time Chat Technical Assistance: Cohort‑specific TEAMS channels that serve as a space for hospitals to ask questions, share resources, and troubleshoot with peer sites, supported with light‑touch guidance from the FQ team.	✔	✔
Facilitation	External Facilitation: Provides 2–4 months of targeted assistance through 3–4 focused sessions (30–60 minutes each) to identify priority barriers and support problem‑solving. An implementation expert guides hospitals using evidence‑based practices, engages leadership when needed, and supplies resources to help sites overcome challenges and sustain STRIDE.		✔

### Measures and data collection

VAMC characteristics reported descriptively include facility complexity, adjusted hospital length of stay, hospital rating, and geography. Facility complexity refers to an organizational measure of hospital-level volume and resources based on services provided. Facility complexity is based on administrative data sources including volume, patient risk, involvement in teaching and research, and breadth of physician specialties. A lower number (e.g., 1a) indicates a higher degree of facility complexity. Hospital adjusted length of stay (medicine) and patient overall hospital rating (inpatient) were obtained from a VA administrative dataset called Strategic Analytics for Improvement and Learning (SAIL) [26], using the measurement from the most recent quarter available relative to VAMCs’ start date. Census Bureau regions were reported to classify the hospitals’ geography.

Data on implementation personnel effort was collected from survey forms among internal VAMC frontline staff and clinicians and external centralized assistance staff on the study team responsible for STRIDE implementation during and after the active implementation period where implementation support (REP & enREP) was provided. VAMC frontline staff and clinicians were surveyed at 3 months, 6 months, and 10 months to report time spent on ongoing implementation efforts. This survey- and attendance-log–based capture of personnel effort constitutes a time-motion approach to activity-based costing. To do so, two Microsoft Forms-based survey items were completed to report estimated time spent since the last assessment point (excluding time spent on ‘Office Hours’ and facilitation activities) for 1) the team member themselves and 2) other members of the implementation team on activities directly and indirectly associated with STRIDE implementation. Where VAMC respondents did not provide estimates for time spent at any of the three data collection points, hours spent were imputed using the median number of hours for the respective timepoint across the rest of the VAMC sites in the respective randomization arm. Further, we used data from attendance logs to estimate internal and external staff members time spent delivering and engaging with facilitation activities and ongoing ‘Office Hours’ centralized assistance. Survey data was merged with attendance records to determine inputs from the local VAMC internal clinician/staff and external study team members to provide implementation support remotely.

Salary and wages were estimated using pay tables for the median national locality-adjusted region (Cincinnati-Wilmington-Maysville) for calendar year 2023 based on position classification and GS-level (a United States federal government employee compensation structure) [27]. Similarly, external facilitator personnel wages were based on median national wages for the role, consistent with the non-location dependent role they perform. As a result, all variation was driven by effort and not location.

### Implementation outcomes

Penetration, or ‘reach’, represents is measured at the patient-level and represents the extent to which STRIDE was integrated within the VAMC as a part of routine operations and was operationalized as the percentage of hospitalizations with ≥1 documented STRIDE walk during months 6–16. This data was abstracted for all patients eligible at the participating hospitals whose hospitalization(s) met inclusion and exclusion criteria for the parent study [28]. Site-level adoption is defined as a binary outcome consisting of whether the VAMC has completed a STRIDE walk with ≥5 patients or not in months 6 through 16 and can include hospitalizations ineligible for calculating the penetration outcome (e.g., patients with documented STRIDE walks who were less than age 60 at time of admission). The 5-patient threshold was selected based on prior work and because it reflects a minimal level of clinical activity needed to show that the program had initiated and because it was consistent with the theoretical conceptualization of adoption or ‘uptake’ as an implementation outcome [29].

### Implementation cost analysis

We used an activity-based, micro-costing approach, used previously by our study team and common in implementation research, to assess the time and resources used to implement STRIDE with REP or enREP support [2, 14]. We specifically assessed direct personnel time associated with discrete REP and enREP implementation activities over the 10-month active implementation effort time period. Implementation costs included labor for VAMC personnel (e.g., planning, hiring, training) as well as participation in off-site implementation support activities (i.e., Office Hours calls and enREP calls). Equipment costs and the design and development of implementation support materials (e.g., a digital toolkit, documentation and mass media templates) were not included in cost estimates and were treated as a sunk cost that do not impact a budget moving forward, as is typical in prior implementation cost analyses [2]. Thus, we conducted this cost analysis from the perspective of a health system leader that makes decisions for staffing care delivery and efforts to introduce new programs to a hospital setting or providing sufficient reimbursement for incentivizing implementation of mobility programs like STRIDE. Costs to patients and caregivers were intentionally excluded, consistent with the health-system decision-maker perspective. Consistent with implementation cost analysis best practices [30] and best practices for specifying and reporting implementation strategies, we sought to estimate cost from the perspectives of different ‘actors’ directly involved with delivering or enacting the implementation strategy [31]. Thus, costs are presented for internal VAMC-level actor effort (e.g., local clinician and staff at the participating VAMC) and external actor effort (e.g., centralized resource effort offered by the study team).

Total hours expended associated with implementation activities were multiplied by the hourly wage. We applied a benefit fringe rate of 30% to salary and wage estimates. We evaluated short-term expenditures; thus, costs were only estimated during the 10-month active implementation period. Estimates are in 2023 US dollars. One-way sensitivity analyses were conducted to illustrate the impact of each implementation activity input parameter on total cost using a 25th percentile and 75th percentile for the distribution of locality-adjusted wages. In doing so, we estimated costs based on a range of labor cost inputs to highlight how varying compensation levels for the same position can influence cost estimates. A deidentified site-level cost and time dataset used for the study is provided as part of Additional file 2.

As an exploratory descriptive analysis, we presented data on STRIDE penetration from the 6-month time point to the 16-month time point across both trial arms (REP & enREP). Due to the small sample size, we calculated a Spearman correlation coefficient to describe the association between cost and penetration overall and by arm. We also reported total costs for VAMCs overall and by arm that met program adoption. To contextualize these summary statistics, we provided descriptive data visualizations for VAMC costs overall and by arm by rates of penetration and adoption. All analyses were conducted in Microsoft Excel and SAS Enterprise Guide version 8.3 (SAS Institute, Cary, NC).

## Results

Nineteen VAMCs were randomly assigned to receive REP only and 16 VAMCs were randomly assigned to receive enREP. VAMCs in each arm were similar across many organizational characteristics including facility complexity, geography, adjusted length of stay, and overall hospital rating; however, REP VAMCs were less likely to report that they had received resources or staffing support to implement STRIDE, but more often reported experience with STRIDE or other mobility program implementation. Additional detail on VAMCs is included in Table 2. Among those VAMCs randomly assigned to enREP, three met the program activity benchmark at 6 months and the program activity benchmark at 8 months and, thus, were not offered additional practice facilitation as part of enREP. The other 13 enREP sites (84%) did not meet the 6 or 8 month benchmarks, and practice facilitation was offered to provide intensified support starting at month six or eight, depending on which benchmark was not met.

**Table 2:** STRIDE VAMC sites by implementation strategy bundle (REP & enREP)

	REP *n* = 19	enREP *n* = 16	All sites *n* = 35
Facility complexity score§, n (%)			
1a	8 (42)	6 (38)	14 (40)
1b	4 (21)	1 (6)	5 (14)
1c	1 (5)	5 (31)	6 (17)
2	4 (21)	2 (13)	6 (17)
3	2 (11)	2 (13)	4 (11)
Adjusted length of stay (medicine), # of days (SD) ^†^	5 (1)	5 (1)	5 (1)
≥4.7, n (%)	12 (63)	10 (63)	22 (63)
<4.7, n (%)	7 (37)	6 (38)	13 (37)
Site reports experience with STRIDE or other mobility program implementation in the last 5 years, n (%)	5 (26)	0 (0)	5 (14)
Site reports receiving resources or staffing support to implement STRIDE, n (%)	2 (11)	5 (31)	7 (20)
Patient overall rating of hospital (inpatient), mean (SD)^†^	72 (6)	72 (8)	72 (7)
Geographic region, n (%)			
Northeast	3 (16)	3 (19)	6 (17)
Midwest	5 (26)	5 (31)	10 (29)
South	5 (26)	3 (19)	8 (23)
West	6 (32)	5 (31)	11 (31)
Rural, n (%)	2 (11)	1 (6)	3 (9)

SD = standard deviation

§Facility complexity level classifies VHA facilities at levels 1a, 1b, 1c, 2, or 3 with level 1a being the most complex and level 3 being the least complex. The model is reviewed and updated with current data every three years. The peer grouping system is based on seven variables relating to patient population, clinical services complexity, and education and research

^†^Calculated based on the Strategic Analytics for Improvement and Learning methods using an adjusted observed minus expected length of stay approach. A lower value generally suggests greater efficiency and reduced risk to patients. One site had an error in their adjusted length of stay placing it in the incorrect strata, analysis was conducted with corrected stratification variable

^†^Overall rating of hospital (higher preferred) is a metric derived from Strategic Analytics for Improvement and Learning (SAIL) measure. SAIL is a system for summarizing hospital system performance within VHA. A facility’s score is percentage of patients responding 9 or 10 on a 0 (worst hospital possible) −10 (best hospital possible) scale their hospital stay

### Cost per site by implementation strategy arm

The median cost of implementation support for REP VAMCs over the 10-month active implementation period was $18,644.58 (Q1–Q3: $14,089–$28,579). Median site-reported time and corresponding costs for implementation activities was $16,796.00 with $1,930.28 of costs associated with Office Hours participation and external support personnel. The median cost of implementation support for enREP VAMCs was $20,006.79 (Q1–Q3: $17,341- $23,083). Median site-reported time and corresponding costs for implementation activities were $17,410.04 with external technical assistance, office hours attendance, facilitation activities and support representing $2,720.18. Using these estimates, we estimate that enREP cost an additional $1,362.22 per VAMC when compared to REP. Additional details on median cost over time and by study arm are reported in Table 3.

**Table 3:** Cost estimates of implementation strategy activities by implementation strategy (Median USD)

REP (n = 19)	Month 1	Month 2	Month 3	Month 4	Month 5	Month 6	Month 7	Month 8	Month 9	Month 10	Total
*Site-level*											
OH Centralized Support	$133	$133	$72	$99	$81	$122	$65	$72	$61	$86	$1,001
Site-Reported	$1,680	$1,680	$1,680	$1,287	$1,287	$1,287	$1,466	$1,466	$1,466	$1,466	$16,796
*External*											
Facilitator OH	$93	$93	$93	$93	$93	$93	$93	$93	$93	$93	$929
**Total**	**$1,860**	**$1,854**	**$1,781**	**$1,459**	**$1,523**	**$1,523**	**$1,624**	**$1,624**	**$1,579**	**$1,676**	**$18,645**
*enREP (n = 16)*	*Month 1*	*Month 2*	*Month 3*	*Month 4*	*Month 5*	*Month 6*	*Month 7*	*Month 8*	*Month 9*	*Month 10*	*Total*
*Site-level*											
OH Centralized Support	$140	$110	$88	$72	$63	$61	$97	$69	$90	$110	$895
Site-Reported	$1,807	$1,807	$1,807	$1,394	$1,394	$1,394	$1,591	$1,591	$1,591	$1,591	$17,410
EnREP	-	-	-	-	-	-	$67	$95	$115	$170	$594
*External*											
Facilitator OH	$93	$93	$93	$93	$93	$93	$93	$93	$93	$93	$93
Facilitator enREP	-	-	-	-	-	-	$58	$46	$70	$81	$302
**Total**	**$2,066**	**$2,019**	**$2,052**	**$1,550**	**$1,636**	**$1,514**	**$1,788**	**$2,414**	**$2,074**	**$2,148**	**$20,007**

Cost estimates reported in 2023 dollars. OH= ‘Office Hours’ referring to a centralized support resource

^a^Virtual Office Hours (OH) were provided monthly using Microsoft Teams to both REP and enREP groups. OH Centralized Support refers to facilitated discussions and webinars on STRIDE implementation, highlight best practices, and promote access to electronic implementation resources and materials. OH Centralized Support also served to facilitate cross-site communication and problem solving across sites. Hours of time costs were calculated based on site-level attendance and personnel resources. Since OH were offered by external research team personnel to sites regardless of randomization arm, they are included in both strategy bundle cost estimate

NOTE: ‘External’ cost estimates refer to personnel costs associated with Durham VA Function QUERI study staff and infrastructure for providing REP & enREP implementation support. For example, providing external practice facilitation via a trained facilitator

Among these enREP VAMCs, the total cost of facilitation varied (median $977 per VAMC; IQR $754–$1,404; range $626–$1,734). This variation was driven primarily by site-level time spent on implementation activities by facility staff and clinicians spent participating in facilitation (median $652; IQR $440–$991; range $282–$1,386). In contrast, the external support reflecting the facilitation team’s effort was comparatively stable (median $325; IQR $279–$372; range $186–$418), because two members of the facilitation team participated in every session, holding the per-session external dose largely constant. Site-level and external facilitation costs for each enREP site are presented in Additional file 2.

### Association between cost and implementation outcomes

**Fig. 2:**
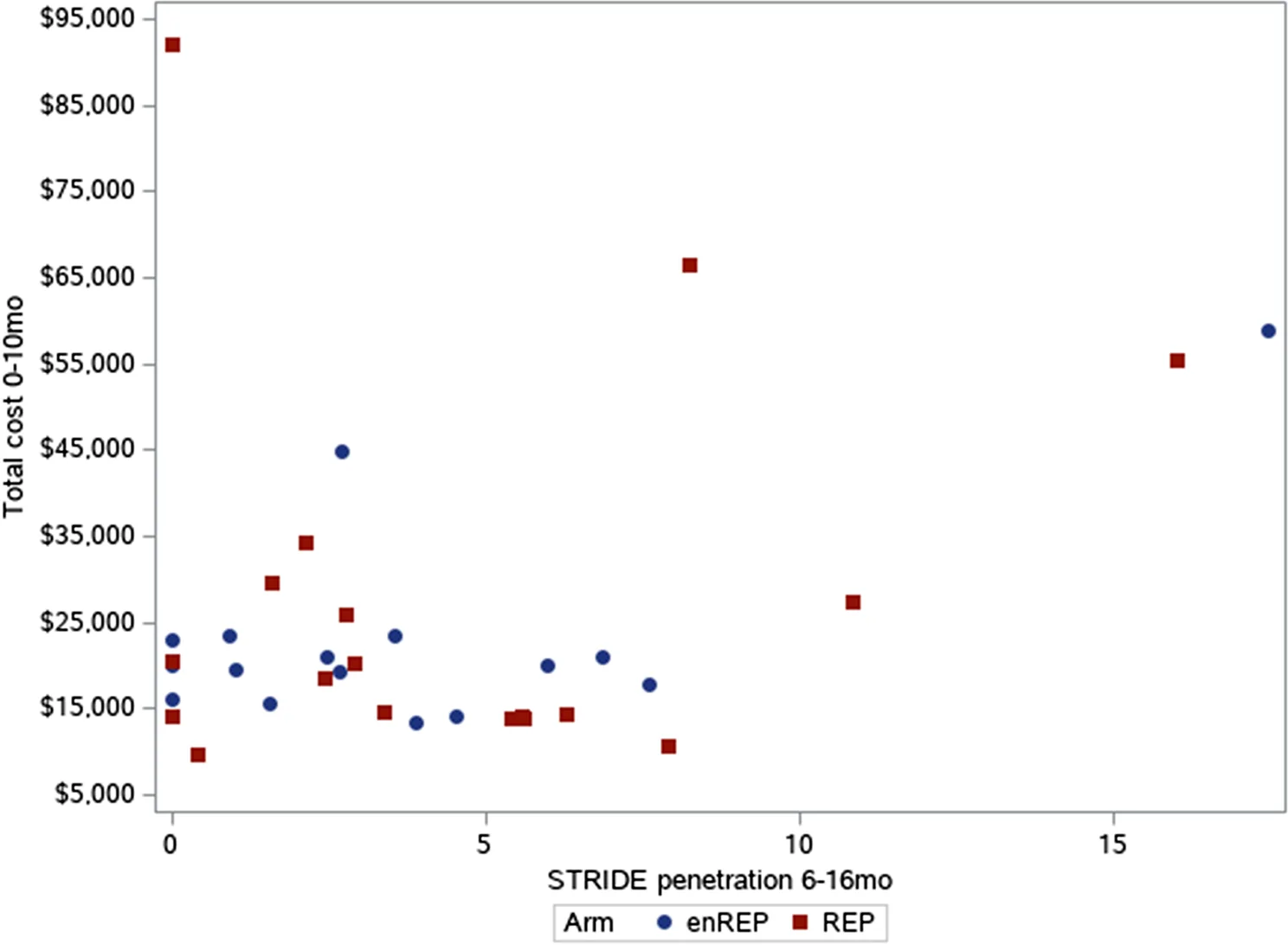
Total implementation cost per site (Median USD) and STRIDE penetration by implementation strategy (REP or enREP). Penetration operationalized the number of hospitalizations with ≥1 documented STRIDE walk during the 10-month observation period (months 6–16)

There was not a strong correlation between total implementation cost and penetration from months 6–16 across all 35 VAMCs represented as a Spearman correlation coefficient (*r* = 0.08). For enREP VAMCs Spearman correlation coefficient between penetration and cost was *r* = 0.06. For REP VAMCs, Spearman correlation coefficient was *r* = 0.02. Figure 2 provides a visualization of VAMC level penetration and cost by implementation strategy arm.

**Fig. 3:**
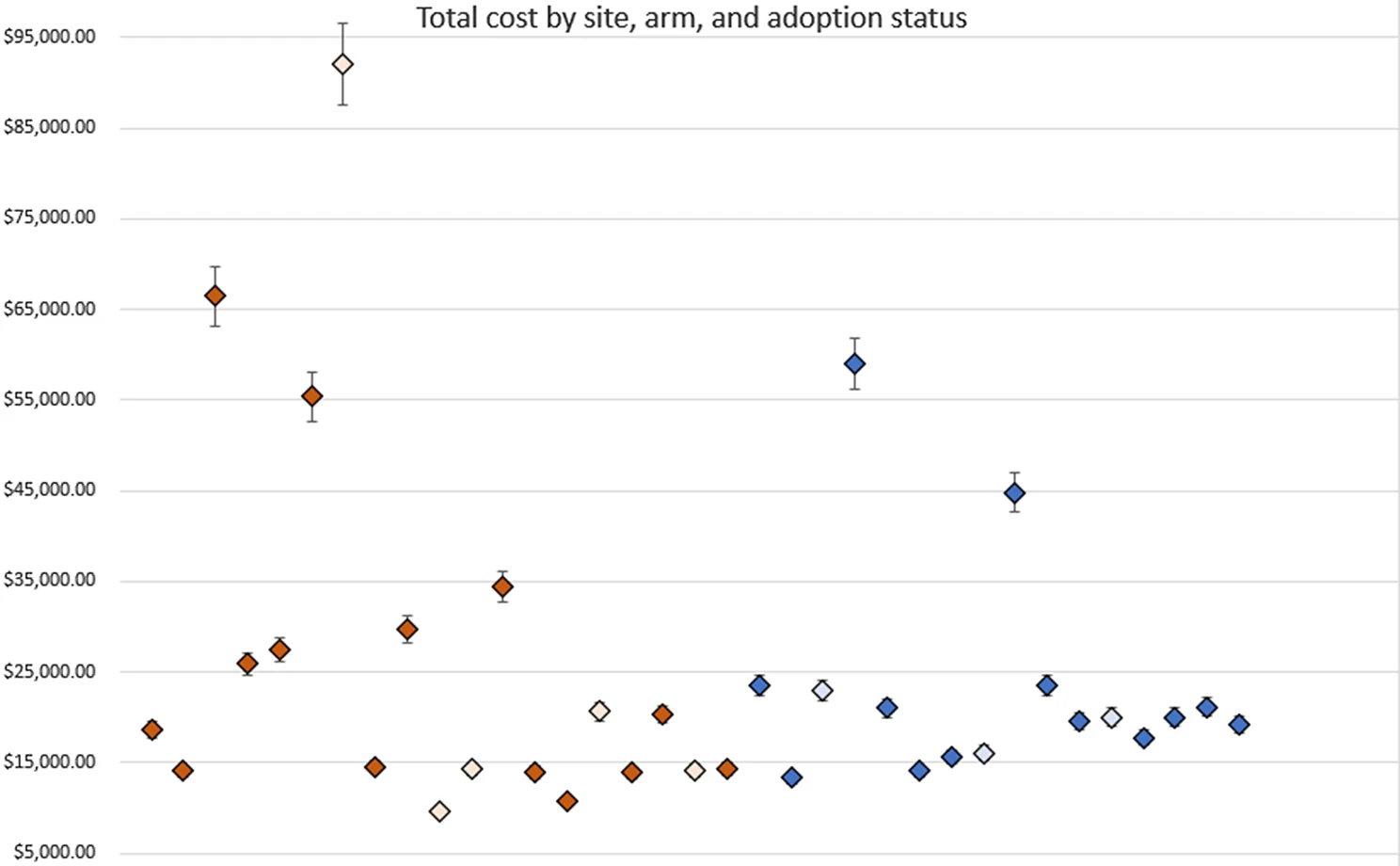
One-way sensitivity analysis illustrating how variation in wages (25^th^, 75th percentile) impacts implementation cost per site (median USD) by adoption status and by implementation strategy (REP or enREP). Red = REP sites; blue = enREP sites. Lighter color shades represent non-adopters. Interval bars represent 25^th^-75th percentile ranges in total costs conducted as part of a sensitivity analysis. Adoption operationalized as a binary outcome, defined as ≥ 5 patients with a STRIDE walk or not for hospitalizations in months 6 through 16

The median cost of VAMCs that adopted STRIDE (*n* = 27 VAMCs) was $20,016 (Q1–Q3: $14,453–$26,679). The median cost of VAMCs that did not adopt STRIDE (*n* = 8 VAMCs) was $18,031 (Q1–Q3: $14,189-$21,204). Among enREP VAMCs that adopted (*n* = 13 VAMCs), total median cost was $20,016 (Q1–Q3: $17,766-$23,485). The median cost of enREP VAMCs that did not adopt (*n* = 3) was $19,998 (Q1–Q3: $18,031-$21,473). For REP VAMCs that adopted STRIDE (*n* = 14 VAMCs), total median cost was $19,488 (Q1–Q3: $14,128-$29,130). The median total cost for REP VAMCs that did not adopt STRIDE (*n* = 5 VAMCs) was $14,213 (Q1–Q3: $14,120-$20,622). In one-way sensitivity analysis, variation in locality-adjusted wages had little impact on median implementation costs (Fig. 3) for either implementation strategy bundle.

## Discussion

We used data from a hybrid type 3 implementation trial conducted at diverse VHA hospitals across the United States to estimate implementation costs associated with two different bundles of strategies of varying intensity to promote implementation of STRIDE, an evidence-based hospital mobility program that has been shown to improve health and utilization outcomes. This analysis was designed to inform decision makers of the cost implications of adopting and scaling STRIDE as part of routine care. We found that implementation costs varied significantly by site in both REP and enREP arms. Notably, we found that enREP cost approximately 7% more than REP. This finding was driven by offering enREP sites practice facilitation activities which constituted approximately 5% of total enREP costs. No significant evidence supported a relationship between cost and penetration overall and by arm. Further, VAMCs that adopted had approximately 11% greater total costs when compared to VAMCs that did not adopt STRIDE. This finding was due to the significant difference in cost between REP VAMCs that did and did not adopt, whereas no significant difference was observed in cost between enREP VAMCs that did and did not adopt.

The relative magnitude and variability of external versus site-level implementation costs have implications for how hospital mobility programs should be budgeted across organizational levels. In our analysis, external costs accounted for roughly 10–14% of total implementation costs and were relatively uniform across sites, while site-level costs accounted for the large majority and showed substantially greater between-site variability (total per-VAMC range $9,666–$92,074).

The timing of costs observed in this trial suggests that there were ‘start up’ costs associated with the initiation of the active implementation effort. Across both arms, we found that implementation costs were greater at the beginning of the implementation period, which aligns with prior research finding costs are higher at the beginning of the active implementation effort due to the upfront effort and infrastructure required to initially adopt a novel EBP. Existing qualitative research on STRIDE implementation sheds light on inputs and effort required for program adoption [14]. Specifically, this required an investment of time from personnel who would assume an ‘advocate’ role to support the program beyond existing clinical duties among other implementation preconditions being met (e.g., documentation templates, referral pathway design) [15, 20]. This finding is not unique to this study as prior literature on early mobility programs recognize start-up costs as an important financial consideration [3]. We found that estimates were sensitive to the type of staff and clinician time spent on these activities given variation in compensation for licensed clinicians (e.g., physical champions or physical therapists) when compared to support personnel (e.g., administrative assistance or physical therapy assistant). This finding emphasizes the importance of efficiently defining roles during, an active implementation effort, particularly the early stages, that match the skills and competencies of available clinicians and staff.

This study builds from existing research assessing STRIDE intervention and implementation costs and represents a contribution to our understanding of the intensity of external facilitation strategies. The authors found STRIDE was a low cost intervention to offer (less than $30 per patient enrolled) and the intensified implementation approach (referred to as CONNECT) was a significant additional investment, costing 68% more than the standardized REP approach but may have achieved greater levels of penetration (i.e., reach) [20]. While CONNECT may have contributed to higher levels of penetration, it was costly and logistically complex given that it required extensive on-site training and workforce development [16]. Offering CONNECT was estimated to have cost up to $94 per enrolled patient, suggesting that, in the short term, implementation may be a more significant driver of cost than intervention delivery [14]. These findings shaped the design of enREP to balance cost and logistical considerations. It is worth noting that facilitation is understood to be a high-intensity activity, but dose has been operationalized inconsistently in prior implementation trials [31, 32]. Kilbourne et al. tested a similar adaptive implementation strategy that intensified to external facilitation for non-responder sites over six months to support uptake of a mental health program in outpatient care settings. Telephonic contact with sites for facilitation activities lasted on average about 30 min, one to three times per month for each facility and led to higher levels of program uptake [25]. Notably, the external facilitation dose we delivered was modest and relatively standardized across sites, whereas the burden borne locally varied substantially; this suggests that the cost and intensity of facilitation are shaped less by the facilitator’s input than by how much staff time each site mobilizes to engage with it. When interpreting these studies together, the results indicate there is a need for intensified implementation strategies to be effective that must be balanced with practical considerations surrounding cost and complexity of implementation activities to ensure they are feasible to offer at scale.

Cost estimates varied significantly by VAMC, over time, and depending on whether VAMCs randomized to enREP did not meet 6- or 8-month program activity benchmarks, thereby receiving intensified support via facilitation. Since the majority (84%) of sites randomized to receive enREP did not meet program activity benchmarks, costs increased as intensified support was offered. Two factors that influenced implementation costs were the staffing models VAMCs used for implementation activities and the level of engagement with external centralized assistance offered via ‘Office Hours’ and, for enREP VAMCs, external facilitation. STRIDE implementation also requires changes to hospital ward practices, logistical processes, and novel relationships and interdependencies of personnel [33–35]. Notably, we observed that ‘Office Hours’ participation reduced after the initial timepoint across sites in both study arms. This may be due to the initial need for external guidance and technical assistance to initially offer the program and then a shift towards effort on site-specific activities associated with expanding reach once the necessary training and guidance has been ascertained from centralized support. In summary, our findings suggest that VAMC personnel engage with centralized technical support at higher levels, driving greater cost, during the initial period of an active implementation effort with a tapering effect occurring after this higher intensity period. Future research should evaluate this trend in implementation costs as sites seek to sustain an EBP.

Staff and clinician time spent engaging with implementation strategy activities impose an opportunity cost to an organization. Previous research describes how implementation and sustainment efforts can fail when sufficient resources are not obligated to support these translational efforts [36]. Further, the main hybrid type 3 trial did not observe a statistically significant difference between penetration, adoption, or fidelity in REP and enREP sites [12]. 81% of enREP VAMCs had adopted STRIDE and 74% of REP VAMCs had adopted but this did not result in a statistically significant difference between arms. There are several plausible explanations. Given the modest between-arm cost differences, enREP implementation support intensity may not have been sufficient to impact implementation outcomes of interest. There may also be opportunity to better target intensified support by proactively identifying sites that may benefit from more labor-intensive facilitation activities. Considering these findings, future research should focus on tailoring and intensity of implementation support based on site-specific barriers. Further testing of an adaptive and tailored approach to promote implementation and therefore improve population-level outcomes, is critical for informing efficient allocation of resources for implementation.

The contribution of this research is based on the recognition that while implementation strategy costs are critical to assess, they are understudied. A 2022 scoping review of implementation strategies used in public health, community, and health care settings found that for multicomponent strategies, the range of costs was $2,244–$11 679 per provider and at the organizational level between $13,546 and $116,911 per setting/site [21] as was assessed in this study. However, authors noted that while the quantity of published studies is increasing, they recommend consistent reporting standards for costs associated with a strategy bundle overall, individual strategies, and by actor that delivers the implementation strategy [21]. Further, the cost analyses that do exist rarely report implementation outcomes associated with the strategies to assess ‘return-on-investment’ to promote the application of comparative economic evaluations. Comparing costs between implementation strategy bundles is vital for providing practical insights into the cost of strategies that decision makers invest in. In the hospital and inpatient care context, there are unique implementation and cost considerations given the skilled and highly compensated workforce with interdependent roles and care responsibilities [20, 37]. Additional research from this trial revealed that VAMCs that adopted (based on the 6 month program activity benchmark) may have differences in organizational characteristics when compared to non-adopters. Specifically, Zullig et al. found that VAMCs that successfully met the benchmark for STRIDE program activity at 6 months were more likely to have lower facility complexity scores, report experience with STRIDE implementation, and report higher levels of organizational readiness, resilience, or implementation climate [38]. Thus, there may be opportunity to efficiently titrate intensity and timing of implementation support based on these characteristics by assessing implementation context proactively, prior to the active implementation effort. Taken together, resource allocation decisions should weigh the potential need for significantly higher intensity implementation strategies than the intensification of facilitation we tested for non-responder sites in this study to overcome barriers like inexperience with the intervention.

Although this study provides valuable insight into the cost of implementing a mobility program for hospitalized older adults, the results should be interpreted through the lens of several limitations. First, the VHA is the largest integrated health system in the United States and, as such, provides a unique continuum of care, especially for older and complex patients. Due to the VHA’s unique service delivery environment, the resource needs for STRIDE implementation may not fully generalize to non-VA healthcare settings. We would anticipate costs to be higher in non-VA settings since, for example, much of the existing implementation materials and documentation infrastructure offered as part of REP are specific to the VA service environment and were not included in the cost estimates. Additionally, not all types of costs are considered by this evaluation. For example, we did not estimate the opportunity cost required of diverting staff effort towards implementation activities and away from usual clinical care or other organizational objectives. Additionally, we treated equipment, materials, and other administrative capacity building as a sunk cost and did not include them in the analysis. This decision may limit the applicability of cost estimates to settings without these materials and infrastructure in place. Further, it should be noted that factors including clinician compensation, pre-existing culture focused on hospital mobility, and centralized support may limit the extent to which findings generalize outside of the VHA service setting. Further, given the time over which the trial was conducted, it is important to note that STRIDE implementation took place during the COVID-19 pandemic which was still disrupting health care delivery and implementation processes, especially in the inpatient setting. Finally, REP sites were less likely to report receiving resources or staffing support to implement STRIDE (11% REP and 31% enREP). Despite the randomization protocols, these baseline differences by arm may have contributed to the findings if enREP VAMCs being more likely to receive resources for delivering STRIDE resulted in greater organizational readiness to more efficiently complete implementation activities. The small sample size of sites within the enREP trial arm also presents potential measurement constraints and limited variability to assess a dose-response relationship with facilitation activities. Despite these limitations, the study is unique in that it leverages a large-scale implementation trial to report cost and its relationship with implementation outcomes.

## Conclusion

In conclusion, we found that intensified implementation support via external facilitation was required by most VAMCs randomized to the enREP arm and was more costly to provide than standardized support offered to VAMCs randomized to the REP arm. We found that all VAMCs required significant investment towards implementation activities, but we did not find evidence of a correlation between total cost and penetration of STRIDE. We also found that VAMCs that adopted STRIDE accrued greater costs than those that did not but this difference was only observed among REP VAMCs. By comparing the costs for varying levels of intensity of strategies and associated implementation outcomes, this study helps provide a multi-level perspective on resources required for STRIDE implementation across a large integrated health system. These inclusive estimates of direct costs over a short-term investment horizon and reporting their association with implementation outcomes provides a framework for using data from implementation trials to provide clarity to decision makers on funding allocations. This information is valuable to ensure implementation strategies are properly resourced and to establish performance benchmarks for their efficiency or ‘return on investment’. Guidance from leadership and operational partners is critical for assessing ‘cost efficiency’ in a flexible way that can be tailored to the nuances of a project. When interpreting implementation costs estimated in this study, as well as previously reported intervention costs [14], it is important to acknowledge that having a STRIDE program has been shown to lower odds of discharge to a skilled nursing facility [12, 39] which reduces health care expenditures and should be considered when considering investments in promoting its use. Future research should further evaluate and compare short-term upfront costs related to implementation with longer-term cost savings and improvements in health outcomes associated with increased reach of STRIDE.

## Electronic supplementary material

Below is the link to the electronic supplementary material.

Supplementary material 1

Supplementary material 2

## Data Availability

The datasets used and/or analyzed during the current study are available as a supplementary appendix and from the corresponding author on reasonable request.

## Abbreviations

CHEERS: Consolidated Health Economic Evaluation Reporting Standards
EBP: Evidence-based program
enREP: Enhanced Replicating Effective Programs
ERIC: Expert Recommendations for Implementing Change
IQR: Interquartile range
OH: Office Hours
POC: Point of contact
QUERI: Quality Enhancement Research Initiative
REP: Replicating Effective Programs
SAIL: Strategic Analytics for Improvement and Learning
SD: Standard deviation
STRIDE: AssiSTed eaRly mobIlity for hospitalizeD older vEterans
USD: United States dollars
VA: Veterans Affairs
VAMC: Veterans Affairs Medical Center
VHA: Veterans Health Administration
